# Experiences of ethnic minoritised young people in a specialist child and adolescent mental health service: A qualitative analysis as part of a mixed methods service evaluation

**DOI:** 10.1177/13591045231208571

**Published:** 2023-10-17

**Authors:** Rachel Sarr

**Affiliations:** Institute of Psychiatry, Psychology and Neuroscience, 34426 King’s College London, UK

**Keywords:** Ethnicity, adolescence, psychological therapy, qualitative methods

## Abstract

**Background:**

There have been several reports of inequalities for ethnic minoritised service users across National Health Service mental health services in the United Kingdom. This research aims to explore the perspectives and experiences of young people from ethnic minoritised groups accessing psychological therapy in a National Specialist Child and Adolescent Mental Health Service in England.

**Method:**

Semi-structured interviews were conducted to investigate how young people perceived their ethnicity and how it was considered during psychological therapy. Nine young people were interviewed, and a thematic analysis was conducted.

**Results:**

Qualitative analysis revealed five themes: (1) adolescence, ethnicity, and identity; (2) ethnicity as a meaningful part of the therapeutic intervention; (3) therapeutic alliance to facilitate dialogue; (4) aversion to ethnicity exploration; and (5) treading lightly: a fine line between sensitive and overly cautious.

**Conclusions:**

The study illustrated the complexity of considering ethnicity in therapy due to conflicting views and preferences and the need for further research.

## Introduction

In 2022, a report commissioned by the National Health Service (NHS) Race and Health Observatory in England found inequalities for ethnic minoritised service users, in terms of poor access, experiences, and outcomes (Kapadia et al., 2022). According to NHS England (n.d.), inequalities are unjust and preventable differences in health among various groups, which can include disparities in life expectancy, overall health and care. The Race and Health Observatory report highlights that these inequalities are not only due to interpersonal racism. They also stem from structural and institutional racism. This occurs when discriminatory practices take place within organisations, such as racist treatment, inadequate recording of ethnic monitoring data and limited availability of interpreting services (Kapadia et al., 2022). In mental health services, inequalities were amongst the largest (Kapadia et al., 2022; Wise, 2022). National Institute for Health and Care Excellence (2018) states the importance of promoting access and health outcomes for Black, Asian and minority ethnic groups, including through the improvement of psychological therapies.

### Child and adolescent mental health services

Race and ethnicity have been shown to impact child and adolescent mental health, with Black and minority ethnic young people being at greater risk for mental health difficulties, compared to their White peers (Hull et al., 2008). Yet, the NHS Race and Health Observatory report (2022) includes an unfavourable review of ethnic inequalities in NHS CAMHS. Discrimination, racism, language barriers and a lack of trust in services were identified as obstacles to accessing services. Young people were reluctant to seek help due to the fear of being misunderstood, having had negative experiences in the past, and services not being culturally appropriate (Kapadia et al., 2022).

### Prior research

Few studies have looked at ethnic minoritised young people’s experiences of psychological therapy. In 2014, Gurpinar-Morgan and colleagues interviewed young people from minority ethnic groups who received cognitive behavioural therapy (CBT) in the NHS. They explored young people’s perceptions of how ethnicity is featured in their relationship with their therapists and its relevance to therapy. Key findings were for therapists “*to demonstrate empathy for clients and an appreciation of how a client’s ethnic background may impact on their presenting difficulties*” (Gurpinar-Morgan et al., 2014, p. 722); to encourage young people to talk about their background during formulation and intervention; to take the lead in bringing up ethnicity and carefully consider the timing, context, and rationale. However, the authors noted that it can be difficult to provide general recommendations for therapists due to the varying views of participants (Gurpinar-Morgan et al., 2014). Due to the limited research exploring ethnic minoritised young people’s experience of psychological therapy, further research in this area is needed.

Other studies have looked at the need for psychological therapy adaptations when working with ethnic minoritised service users. Therapists need to consider social contexts, beliefs about distress and help-seeking, coping mechanisms, and experiences of racism as they are all elements that can impact presenting difficulties, access to help, engagement and outcomes (Cantor-Graae & Selten, 2005). Sue and colleagues (1992) spoke of a culturally competent therapist when the following three elements are demonstrated: cultural awareness and beliefs, cultural knowledge and cultural skills. One could argue that these are never completely attained and should continuously be developed to work with service users’ diverse ethnic backgrounds. Changes can also be implemented on the intervention level. According to Sue et al. (2009), this can include delivering treatment in the language of the service user or ensuring the therapist and service user match ethnically and culturally. Beck (2016) also advocated that CBT could be culturally adapted (CA-CBT) or culturally sensitive (CS-CBT). In CA-CBT, CBT is adapted for specific ethnic groups and specific presentations. While the traditional CBT techniques remain, language and examples are culturally specific, and there is a greater emphasis on cultural practices, migration history, and discrimination (Naz et al., 2019). In CS-CBT, therapy is only adapted on a case-by-case basis, when needed, to include discussions on ethnicity and culture (Naz et al., 2019). While prior research states the need for adaptations when working with ethnic service users, it was reported that many therapists do not feel confident asking about the ethnicity of the service users they work with for fear of offending or making mistakes (Naz et al., 2019). Thus, this paper aims to address how therapists can consider this important aspect when working with young people.

### Aims

This study aimed to assess ethnic minoritised service users’ views of their ethnicity and explore their experiences of psychological therapy, precisely the consideration of their ethnicity in their care.

The terms ethnicity, race and culture are often used interchangeably. This paper focuses on ethnicity as it is a broader social construct encompassing crucial aspects of one’s identity. Ethnicity is defined as the “*definition of oneself in relation to a range of factors including language, geographical origin, skin colour, political preferences and religious and cultural practices*” (Beck, 2016, p.8 in Transcultural Cognitive Behaviour Therapy for Anxiety and Depression: A Practical Guide).

## Method

### Setting

This study was conducted in an NHS National and Specialist Child and Adolescent Mental Health Service located in England. This service provides diagnostic assessments, consultation, training, supervision and treatment for depression, anxiety and trauma, mainly in the form of CBT. The service users are children and young people who have previously received unsuccessful treatment elsewhere and/or need specialist input.

The research obtained Clinical Audit Ethical approval from the NHS Trust CAMHS Governance (CAG Clinical Governance/Audit Committee) on 07/01/2022, and Information Governance approval from the Information Governance Steering Group on 06/01/2022. Participants were provided with information sheets and gave their written informed consent to participate, under procedures approved by the CAG Clinical Governance/Audit Committee and the Information Governance Steering Group. Parental consent was also sought for participants under 16 years old.

### Design

The study adopted a mixed-method approach but this paper focuses solely on the qualitative component. This involved conducting interviews with young people to gain insight into their perspectives regarding how their ethnicity should be taken into consideration during therapy. The purpose of these interviews was to gather information on ways to enhance the quality of care provided by the service.

### Participants

Sampling was purposive, where the researcher and team members sought out participants. This included young people whose difficulties allowed for being interviewed and those meeting inclusion criteria. The inclusion criteria were: having been discharged from the service in the last 18 months following at least 3 months of therapy or currently being seen in the service for at least 3 months of therapy; identifying as non-White or non-western; and consent given to be contacted for research purposes.

Recruitment was prepared by compiling a list of eligible ethnic minoritised young people, with the help of clinicians and the screening service users’ records. Several young people (*n* = 12) were not eligible for the study due to having been seen for therapist-supported internet cognitive therapy and not having given consent to be contacted for research. Eligible young people were sent an information sheet and offered a phone call. Parents were contacted for young people under 16 years old. Some young people and parents did not reply or chose not to take part (*n* = 6), and reasons included having no interest in the study, being busy and not wanting to think about their time in the service. Nine young people met the inclusion criteria and provided informed consent.

Participants, aged 12–19 years old at the time of the interviews, had been assessed and accessed CBT in the service. They self-identified as Black Caribbean (*n* = 5), Mixed White and Black Caribbean (*n* = 3), and Asian British (*n* = 1).

### Interviews

An interview guide was developed based on existing literature (Adewole & Atkins, 2022; Gurpinar-Morgan et al., 2014; Rathod et al., 2020), to answer the following research questions:

• How do young people from ethnic minoritised backgrounds conceptualise their ethnicity?
• How do they experience psychological therapy, specifically the consideration of their ethnicity?
• What factors could benefit young people from ethnic minoritised groups during psychological therapy?

The guide was revised following consultation with clinicians in the service. Additionally, it was piloted with two young people to improve the clarity of the questions and ensure the language was developmentally appropriate. The supplementary material includes a copy of the interview questions.

Interviews were conducted online on Microsoft Teams. The interview guide was adhered to, ensuring the research’s aims were addressed, but discussions were also guided by the participants.

### Data analysis

Interviews were analysed using Braun and Clarke’s reflexive thematic analysis (2006) and the steps were as followed:

1. Familiarisation with the data – Interview transcriptions were read twice by the researcher while noting initial reflections.
2. Generating initial codes – This was conducted using Nvivo. There was no coding framework and coding was conducted by one person, allowing for researcher subjectivity as an inherent part of knowledge production (Braun & Clarke, 2006).
3. Searching for themes – Initial codes were grouped according to their relevance to each other, with possible theme names. These initial themes were created using an inductive approach, without a pre-existing coding frame or preconceptions from the researcher. Codes and themes were identified at a semantic level.
4. Reviewing themes – The codes within each initial theme were reviewed against each other to consider coherence. Themes were then reviewed against the original data set.
5. Naming themes – The themes were renamed to best capture the essence of the codes and data they highlighted.

Within the reflexive thematic analysis method, the researcher’s subjectivity is an analytic resource used for data interpretation (Braun & Clarke, 2020). Thus, researchers must reflect on their positionality. The researcher identifies as being of mixed ethnic background and recognises that this individual context cannot be removed from the study and may impact the interpretation of findings.

## Results

**Figure 1.**
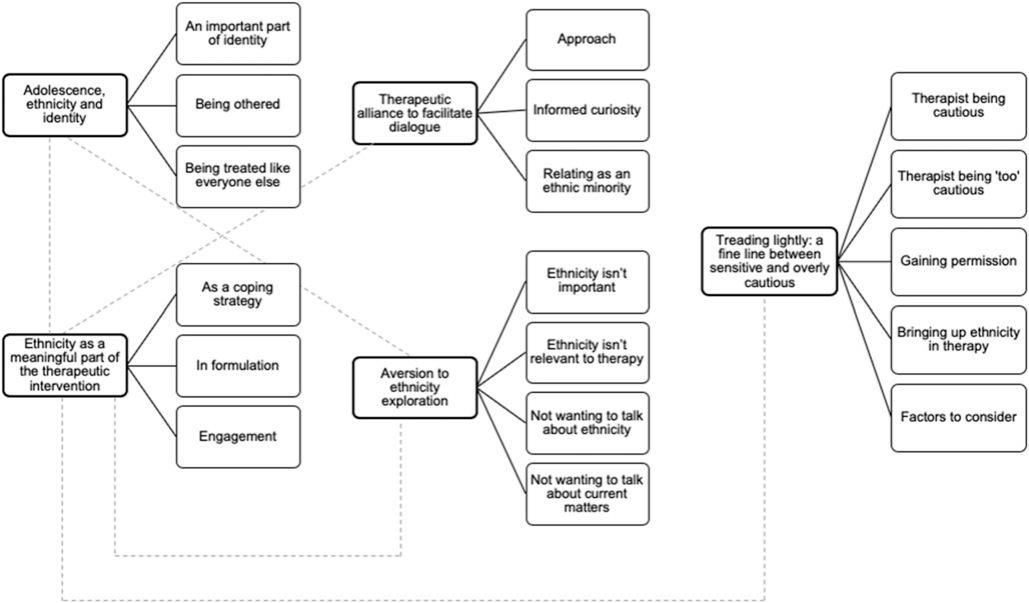
Thematic map highlighting themes, subthemes and the relationship between themes.

As shown in Figure 1, the analysis generated five main themes, which will be discussed in the following sections.

### Adolescence, ethnicity, and identity

The first theme relates to understanding the importance young people attribute to their ethnicity, which in turn can influence its consideration in therapy. All participants gave their ethnicity, and many chose to specify their family backgrounds, their birth country and/or nationality. This highlights the varied meanings ethnicity can have. Young people attributed varying importance to their ethnic background. For some, ethnicity was an important part of who they are.

It makes me who I am because if, for example, both my parents were English, I think I would be a completely different person to who I am. (P9)

I think that it's a part of you […] it’s what makes you different […] it’s something personal to you. (P3)

Ethnicity relates to a shared cultural heritage, history, origins, roots, and family, which can help shape one’s identity. Ethnicity can also impact the way young people express themselves, and their likes and dislikes, such as food or music. Thus, at an age of identity development and formation, ethnicity seemed to be a contributing factor for some participants.

It has cultural roots, historical roots […]. So, it is important to [how] I represent myself as well. (P2)

It represents kind of who I am and my family and […] seeing things on the news and [how] Black people are viewed and represented and stuff. Kind of important, I guess to challenge that narrative. (P7)

I would enjoy different things. And like different music, different food, different atmospheres, I just think […] everything about me would be a lot different ‘cause I like express myself a lot through my ethnic background. (P9)

In contrast, some young people reported not identifying with their ethnicity and described it as not being important to them. For two participants, there may have been a dilemma of belonging to an ethnic minoritised group but not fitting in with peers or parental expectations. One of the participants explained that due to not feeling part of their culture, and not identifying with their ethnic background, speaking about their ethnicity gives them a sense of being “othered”.

It’s not important to me. (P5)

I don’t feel part of my culture. […] I feel like if it’s brought up loads, because I feel very English, I feel a bit ‘othered’. (P6)

Participants also emphasised wanting to be treated like everyone else, placing the focus on equality rather than equity.

I appreciate it when therapists they don’t look at me as oh, I’m a Black person, they just look at me as a person so anybody can have that treatment, anybody could have that advice given, it doesn’t have to be tailored so it’s like, oh, you’re Black so I have to say this or I can’t say that, you know. (P2)

Don’t treat them differently just because they’re from a different country or religion. (P8)

### Ethnicity as a meaningful part of the therapeutic intervention

Participants highlighted three ways ethnicity can be considered in a psychological therapy setting. Firstly, it can help with getting to know the young person and establishing a therapeutic relationship during the early therapy stages. However, it could also contribute to the “othering” feeling described previously, and as highlighted by Participant 4, young people may be uncomfortable and less likely to share early on.

When starting any relationship with their therapist, kind of, talking about [ethnicity]. (P7)

I guess you could say in the beginning when we were getting to know each other and stuff. If there was probably conversation about like where are you from? […] Not necessarily just where I’m from, background wise […]. (P9)

I don’t know if I’m asked that I would answer. (P4)

Secondly, therapists can consider ethnicity when formulating. For many young people, ethnicity influenced their mental health. For example, Participant 2 described having to suppress their distress because mental health can be dismissed in their culture. Participant 9 noted that their religion impacted some of the beliefs they developed towards life events.

If you’re a Caribbean and you’re Black as well, mental health isn’t like a thing, and especially if you’re young as well as like, oh, what do you have to worry about? What are you sad about? […] I feel very like, uncomfortable, very self-conscious to talk about any form of mental health problems that I have because of my race 'cause like they think, […] oh, you’re a strong Black woman like an independent, […] it’s like, oh, I don’t always feel strong, I don’t always feel independent, sometimes I feel helpless and vulnerable. (P2)

I think of the way I’ve been brought up. I guess my grandparents being brought up and then being around me and seeing how I’ve been, well, not really coping with my mental health. It was quite difficult in that retrospect and I did speak with my therapist about it. (P7)

We spoke about the fact that I was a Christian and how, like my beliefs could like be adding towards the way I feel about what happened and what goes on in my life. (P9)

Finally, ethnicity can be considered a coping strategy in two manners. On the one hand, it could be helpful. For example, a participant explained that they were asked to imagine they were in their home country during safe space imagery, which helped them feel better. Another had discussions with their therapist about the Bible and its impact on them. However, for one of the participants it was about realising that religion could prevent them from feeling better. Thus, ethnicity as a coping strategy can also be about realising how it may hinder well-being.

I guess with me I’m a Christian and there is a lot of different viewpoints and opinions that I have in comparison to like what’s being put in the Bible for example, and we kind of thought of that and how that’s made me feel. And yeah, I definitely feel that my therapist was very understanding or how it kind of impacted me. (P7)

‘Cause like I’m Christian or whatnot […] like my religion is probably an obstacle for me in terms of mental health because I don’t actually know how to feel about it. (P2)

### Therapeutic alliance to facilitate dialogue

While discussions related to ethnicity in therapy were meaningful for some participants, the therapeutic alliance was shown to be crucial in facilitating the dialogue. Participants described a therapeutic approach to ease what is sometimes a complex conversation. Being understanding, validating, respectful and considerate were valued by young people.

Making sure that I feel valued and respected. Like you know from where I come from that, doesn’t like, I’m not discriminated or anything like that. (P3)

I guess I would say that my therapist was really understanding and considerate of how, I guess with the views of my family around their background on how they’ve been brought up. (P7)

She was so willing to talk about it and genuinely seems concerned that had happened to me and upset me like beyond just, patient and like staff member it was more like a general upset feeling for another human being? It was kind of thing where you had to be there to understand. (P9)

Informed curiosity is also important. A participant highlighted that therapists should gain knowledge of their service users’ ethnicity ahead of sessions while acknowledging that it can be a broad subject. Therapists showing curiosity and asking questions was also crucial, as well as recognising and acknowledging when they were wrong.

For someone who is Christian and the therapist is Christian then I think it’s not important for them to do a lot of research into or learn. But if they’ve got a new patient that is, uh, a Muslim. […] I think it would be nice to for them to be able to notice that patient is from whatever minority, get some knowledge on what they would go through […] I’d say because ethnicity is such a broad subject that it's hard to encapsulate it all in just. Like an hours’ worth of reading or something like that. So just knowing a little bit I think goes along way. (P9)

I think there was a point in time where one of my [family members] had a funeral and it’s like there’s always like in Nine Night funeral and [Therapist] didn’t have a clue that was and I was surprised she did pay interest and would seem to like be happy to learn something new. (P9)

We’ll talk about Jamaica and [Therapist] seemed very understanding and like curious to [know more]. (P4)

If they want to say something that wasn’t entirely correct. And the person that will tell like. Just to correct them and actually tell them what it is correct. (P9)

One participant mentioned their experience of having a therapist who was also from an ethnic minoritised background, which allowed them to relate to some of their experiences.

When like a therapist […] can relate to your problems as like an ethnic minority or like they understand. […] you can still be the person you want to be and, um, that is really nice to hear from somebody else who is also like a ethnic minority. (P2)

### Aversion to ethnicity exploration

While ethnicity can be interwoven into the therapy, this should not be done systematically. Some young people said that they did not want to discuss it with their therapist, due to ethnicity not being important to them and the feeling of “othering”. Similarly, with using the therapy space to discuss related socio-political matters, a participant explained they did not want to do so with their therapist. They said it would not come from a genuine place, could feel tokenistic or it would have been outside of therapy already.

I would prefer not to talk about [ethnicity] […] unless I bring it up. (P5)

If it’s not brought up, it feels a lot more normal for me. Unless obviously there’s a reason that it has to be brought up and it’s, you know, it makes sense. But when it’s brought up for sort of no reason, it does feel a bit ‘othering’ I guess. (P6)

I didn’t really want to talk about [Black Lives Matter] because I thought like if-if somebody else was going to speak to me about it I feel like it wouldn’t, it wouldn’t sound genuine and they would have thought like oh, surely I have to talk about it because, you know, she looks Black or she, you know, is like a part of that community, so she probably wants me to talk about it, but it’s like, no, like what happened was terrible, but not, not every Black person wants to speak about that, especially if they’ve had the conversation like multiple times already, or they already know this, so there’s no point of speaking about it all the time. (P2)

Some participants questioned the utility of discussing ethnicity in therapy. Is it necessary? Is it relevant to the therapy? This suggests that a conversation around ethnicity is helpful if it is relevant to the presenting difficulties and/or the agenda items, or if it has been established that it is important to the young person.

I don’t think it’s necessary for this type of therapy, but if it was like for something like identity or like having like, um, gender or some form of dysphoria, then I guess that would have to come up [more]. (P2)

I don’t know why it needs to be brought up. I think maybe in today’s society, yes, because there’s still things that go on that […] but. I don’t really see like I don’t see why that all needs to be brought into it. If I’m completely honest. […] I don’t see what that has to do with a counselling session for trauma. I don’t see how they correlate. (P3)

### Treading lightly: A fine line between sensitive and overly cautious

Previous themes illustrate the complexity of considering ethnicity in therapy with young people. Some participants wanted it to be discussed and others did not. While therapists should bring up ethnicity with caution, some participants noticed their therapists being “too cautious”. Thus, the discussion can be brought up sensitively but directly.

[When] they understand so they tread lightly on, on certain things or, or they don’t have to be like avoidant or complacent. (P2)

[Bringing it up by] not too going around the bush, I guess. [Reading] someone’s behaviours to certain questions, you know, learning when to use different tones or stuff. (P4)

When they’re talking to you and they’re trying to be really careful, really sensitive about it, I understand. That’s their job. It just makes me feel like. It makes me feel like it’s, they’ve gotta watch what they say or else they might say something really horrible […]. But that’s how they’re going on because they’re being so sensitive about it, because they’re being so careful about it. (P3)

The thing that I didn’t like is that when I was talking about, um, how I am as a person, like Black or whatnot and religious and stuff, where like it was like, like you know that voice that people use like it’s like, oh, you okay? It’s like a very baby voice and I felt like, are you doing this because, like, I’m talking about something like this or, like, do you think, like, I’m like, I’m stupid or what not. (P2)

With such varied views on the topic, therapists could gain permission from the service users they work with. Depending on service users’ preferences, therapists can then choose to bring up ethnicity if it is of importance. While some young people preferred the topic brought up early on by the therapist, others preferred to bring it up themselves. Thus, therapists should not be afraid to initiate the conversation, but also to provide the space for it, even if it is not on the planned agenda.

Maybe at the beginning and so they know whether to approach it later on in their sessions and what’s appropriate to talk about. And what’s not so. (P7)

I think maybe approaching it in a more relaxed way where maybe pre, even pre-warning, the person like we’re going to have a conversation about this. Let me know if you feel uncomfortable at any time. And then. Get into it. (P3)

Like “Can I ask you this question” or you know? (P4)

It was really nice to know that she would ask even if it wasn’t [important to] me. (P4)

I think it would be nice for someone to bring it up to you ‘cause like so it shows that someone is actually genuinely concerned and cares to hear what you have to say about. You see, so yeah, straight up and straight forward. (P9)

I’d rather [therapists] bring it up. (P8)

It was completely off topic to what our session was about. That means a lot and it got brought up purely because we’re talking about it. And she didn’t kind of say get back to let’s get back to like what we’re here for. (P9)

There is no recommendation as to how therapists should bring up ethnicity and related matters. For participants, the preference was for an ordinary conversation and not in a tokenistic manner.

It’s not like if it’s like an offensive question or it’s like a taboo. […] you don’t always have to bring up current affairs and stuff, I feel like it would only be appropriate is if like they brought it up or if like they wanted to discuss it, but yeah. For example like if I was like, um, Southeast Asian like don’t, don’t make it directly oh I’m going to talk about this because you’re Southeast Asian or I’m going to speak about this because you’re Black, like you don’t have to do that, it’s like as if you’re talking to like a normal person, you wouldn’t just randomly go up to like, to like a White person and then start talking about like the Queen, would you? (P2)

It’s not something that I get funny about or feel sensitive talking about so I get it could literally be mentioned anytime really. (P3)

I don’t really appreciate the questions just being brought up for no reason. (P6)

Therapists should be aware of racism and micro-aggressions, common amongst ethnic minoritised groups, and therapy can be the space to discuss these. As opposed to dismissing this experience, a therapist recognising and naming experiences as racism was appreciated by a participant.

There was an occasion where a boy like came up. I mean, just showing pure verbal abuse. […] I felt scared bringing such a delicate subject […] [Therapist] seemed to know how to deal with it well. […] I had dealt with this kind of situation […] like I said it to my mum, to my family. They kind of didn’t see it as that serious. […] they kind of just dismissed it, but once I spoke to [Therapist], I realized how serious it was. I mean, I’m half Black half White. But still somewhere along the line someone would be racist towards me and it’s nice just to know that there’s a big foundation of people that doesn’t just see us as patients that they’re able to identify that, like different people, have different beliefs and should be dealt with differently. I think it’s a nice thing. (P9)

Therapists should be mindful of making stereotypes when working with service users from ethnic minoritised groups.

You know, you’ve got like, oh, say you’ve got young boy, young Black boy and he dresses a certain way. That’s purely because he’s comfortable. But stereotypes say it’s because he’s a road man. So I think just being mindful of that, not making him feel like he’s doing anything wrong? Just not making him feel awkward. Not making him feel like everyone is watching him, but that goes without saying. (P3)

The fact that I’m from Jamaica. If [Therapist] was just to assume that my family smokes weed because a lot do just like stereotyping, or obviously, if there’s only negativity towards […] anything negative about someone else. (P9)

## Discussion

Ethnicity was important to many of the young people interviewed, shaping their mental health and identity, and contributing to their expression of self and interests. For some, ethnicity was not considered important, and they did not identify with it. Young people can feel judged for not conforming to the expectations of their ethnic group (Gurpinar-Morgan et al., 2014). The importance to all was being treated like everyone else. Thus, at the start of therapy, therapists could ask every young person about the importance of ethnicity to them. This could be done in a normalising way to avoid “othering” (i.e., “I ask this to all young people I work with”).

Considering ethnicity in therapy with young people is a complex task as some want it spoken about, and others do not. Every individual has a unique history but people from ethnic minoritised backgrounds are especially influenced by additional factors such as migratory stressors, ethnocultural and religious beliefs, and triggers including racism and discrimination (Rathod et al., 2019). This study highlights the need for therapists to be person-centred and attuned to young people’s needs (Adewole & Atkins, 2022).

While therapists could explore the importance of ethnicity in the early stages of therapy, they should remember that some young people may not open up this early on and revisit the subject at a later stage. Previous research has shown that a similar ethnicity between young person and therapist was not found to be required for effective work (Gurpinar-Morgan et al., 2014). Yet, a participant in the current study, expressed that sharing the same ethnicity with their therapist enabled them to relate, thus potentially making the conversation more comfortable. Nevertheless, whether the same ethnicity is shared, discussing similarities and differences in their backgrounds is recommended (Gurpinar-Morgan et al., 2004). If ethnicity is deemed not important, therapists must be cautious to not ask too many questions, or make assumptions, which could reinforce the feeling of being different (Gurpinar-Morgan et al., 2014). Some young people questioned the utility of bringing up ethnicity in therapy. Only if it is relevant to the presenting problem and/or important to the young person should it be considered as part of the engagement, assessment, formulation and intervention. If it is discussed, the therapeutic relationship is crucial. An understanding, respectful, curious, and knowledgeable therapist facilitates the conversation and validates difficult experiences. To create a safe space for young people, therapists should adopt a tactful approach: refrain from avoiding the subject, bring it up in a manner which is not tokenistic and allow time to discuss it. They should also be aware of their own biases when working with minority ethnic groups. This is especially true given the breadth of differences in ethnic minoritised groups and subgroups. Thus, specific training and supervision are needed for clinicians to best approach working with young people from ethnic minoritised groups (Rathod et al., 2019).

### Limitations

When considering the broader implications of these findings, it is crucial to proceed with caution given the limitations of the sample size and homogeneity. Although the findings represent varied views and experiences, recruitment was purposive as the team helped choose participants and the sample size was small. There was also an overrepresentation of Caribbean and England-born participants. Nevertheless, the viewpoints and lived experiences of ethnic minoritised young people accessing mental health services are important.

The responses collected may be biased by self-report and social desirability. All young people were informed of confidentiality principles and made aware that their therapists would not know what was said during the interviews. However, only a few young people reported negative experiences. While this may be a coincidence, it could also be that they did not want to upset their past or current therapist. Young people who declined in taking part in the study may have had valuable information to share. Additionally, the study was cross-sectional, providing a one-off view of experiences. This meant that the participants were at varying stages of their therapy journey and did not have the same number of sessions or relationships with their therapists. A longitudinal method may have illustrated fluctuations in experiences.

### Implications and future studies

Findings provide insights regarding the appropriate therapist approach to address ethnicity in psychological therapy, thus improving the experiences of ethnic minoritised young people. It may be helpful for therapists to ask young people about the significance of their ethnicity and how it pertains to their therapy sessions. This can help determine if it should be incorporated into the formulation and intervention. It is recommended that therapists inquire early on in treatment and revisit the topic during the therapy. Additionally, findings support the current model of CS-CBT. It is beneficial to address ethnicity after building rapport early on in therapy and therapy should be adapted as needed, rather than systematically.

Further research using a larger and more diverse sample may collect more varied experiences. Differences between therapist and service user were briefly touched upon when a participant reported being able to relate to their therapist also being of an ethnic minoritised group. It may be valuable to further explore if ethnic minority matching or dissimilar characteristics matter for young people and how it impacts the therapeutic relationship. It may also be interesting to explore therapists’ views on the matter and how they view their self-awareness, knowledge, and skills around ethnicity. Finally, although religion was briefly mentioned by one participant, this study did not explore other aspects of diversity. In future research, intersectionality could be explored to recognise and address the interrelatedness of social identities and how this might be considered in therapy with young people.

## Supplemental Material

Supplemental Material - Experiences of ethnic minoritised young people in a specialist child and adolescent mental health service: A qualitative analysis as part of a mixed methods service evaluation Click here for additional data file. Supplemental Material for Experiences of ethnic minoritised young people in a specialist child and adolescent mental health service: A qualitative analysis as part of a mixed methods service evaluation by Rachel Sarr in Clinical Child Psychology and Psychiatry.
