# Supplementary material for: Experiences of ethnic minoritised young people in a specialist child and adolescent mental health service: A qualitative analysis as part of a mixed methods service evaluation
Source: Clin Child Psychol Psychiatry. 2023 Oct 17;29(1):127–40. doi: 10.1177/13591045231208571 (PMC10748456; doi:10.1177/13591045231208571)
Supplement: Supplemental Material - Experiences of ethnic minoritised young people in a specialist child and adolescent mental health service: A qualitative analysis as part of a mixed methods service evaluation [file sj-pdf-1-ccp-10.1177_13591045231208571.pdf]

The interview started by checking the age and ethnicity of the participants. Then, they were asked if they had heard of the word "ethnicity" before and what it meant to them. They were given the following definition to ensure comprehension "*It means a group to which people belong, which can depend on where people are from. The group can also form due to similar origins, culture, religion, traditions, customs etc. For example, I'm from* [interviewer nationality] *and I consider myself to be* [interviewer ethnicity]".

The interview included some closed questions for the quantitative survey of the study. This was followed by open-ended questions to explore views and experiences in the qualitative study:

- Why is your ethnicity important or not important to you?
- If you were to be asked, when would you prefer to talk about your ethnicity?
- How would like it to be brought up?
- What would you want your therapist to do or keep in mind when talking about ethnicity?
- What was the best part about how your ethnicity was discussed?
- What didn't you like about how your ethnicity was discussed?
- Is there anything you think therapists should do differently when working with young people from a minority ethnic group?

All questions included examples for clarification and/or prompts when necessary (e.g., "*Can you tell me more?*"). An adapted version of the interview guide was created for under 13-year-olds with some of the questions omitted and the language simplified.
